# Vitamin D binding protein in psychiatric and neurological disorders: Implications for diagnosis and treatment

**DOI:** 10.1016/j.gendis.2024.101309

**Published:** 2024-04-15

**Authors:** Ling Li, Bing Han, Yan Kong, Gaojia Zhang, Zhijun Zhang

**Affiliations:** a Research Institution of Neuropsychiatry, School of Medicine, Southeast University, Nanjing, Jiangsu 210009, China; b Department of Neurology, Affiliated Zhongda Hospital, Nanjing, Jiangsu 210009, China; c Department of Pharmacology, Jiangsu Provincial Key Laboratory of Critical Care Medicine, School of Medicine, Southeast University, Nanjing, Jiangsu 210009, China; d Department of Biochemistry and Molecular Biology, School of Medicine, Southeast University, Nanjing, Jiangsu 210009, China; e Brain Cognition and Brain Disease Institute, Department of Mental Health and Public Health, Faculty of Life and Health Sciences, Shenzhen Institute of Advanced Technology, Chinese Academy of Sciences, Shenzhen, Guangdong 518055, China

**Keywords:** Neurological disorder, Polymorphism, Psychiatric disorder, Structure, Vitamin D binding protein

## Abstract

Vitamin D binding protein (VDBP) serves as a key transporter protein responsible for binding and delivering vitamin D and its metabolites to target organs. VDBP plays a crucial part in the inflammatory reaction following tissue damage and is engaged in actin degradation. Recent research has shed light on its potential role in various diseases, leading to a growing interest in understanding the implications of VDBP in psychiatric and neurological disorders. The purpose of this review was to provide a summary of the existing understanding regarding the involvement of VDBP in neurological and psychiatric disorders. By examining the intricate interplay between VDBP and these disorders, this review contributes to a deeper understanding of underlying mechanisms and potential therapeutic avenues. Insights gained from the study of VDBP could pave the way for novel strategies in the diagnosis, prognosis, and treatment of psychiatric and neurological disorders.

## Introduction

Vitamin D binding protein (VDBP), originally referred to as “group-specific component” (Gc), is a polymorphic serum protein that exhibits a wide array of functions.^1^ It was named as such based on its ability to bind to and transport vitamin D analogues.^2^ Following the identification of macrophage-stimulating properties, VDBP was subsequently referred to as macrophage activating factor.^3^ Nevertheless, it is currently referred to as VDBP. Research has witnessed remarkable and substantial progress in comprehending its numerous physiological, biochemical, and molecular functions and characteristics in the past few years. Extensive investigation has provided insight into its contributions to liver disease,^4^ reproductive health,^5^ and malignant tumors,^6^ resulting in comprehensive summaries of these roles. Nevertheless, the systematic delineation of VDBP in psychiatric and neurological disorders remains pending.

This review aims to fill this gap in knowledge by providing a thorough overview of the role of VDBP in psychiatric and neurological disorders. Given its abundant presence in serum and its multifunctional nature, VDBP has emerged as a potential key player in these complex conditions. By summarizing the latest research findings on the structure, polymorphisms, and distribution of VDBP, this review offers valuable insights into the potential involvement of VDBP in various psychiatric disorders such as depressive disorder and schizophrenia, as well as several neurological disorders such as Alzheimer's disease (AD), Parkinson's disease (PD), multiple sclerosis (MS), amyotrophic lateral sclerosis (ALS), epilepsy, restless legs syndrome (RLS), and migraine. By elucidating the interactions between VDBP and these disorders, this review seeks to shed light on possible molecular mechanisms that may underlie disease progression and pathogenesis.

## General characteristics

### VDBP genetic structure

The human VDBP gene, alternatively referred to as the *GC* gene, is situated on chromosome 4 at position 4q11-q13. It consists of a 5′-flanking region spanning 4228 base pairs and a 3′ flanking region spanning 8514 base pairs.^7^ The gene consists of 13 exons that code for 474 amino acids, which include a shortened 16 amino acid leader sequence. The *GC* gene is situated near the genes coding for albumin (*ALB*), α-fetoprotein (*AFP*), and afamin (*AFM*); and the family is connected in the sequence centromere-3′-GC-5′-5′-ALB-3′-5′-AFP-3′-5′-AFM-3′-telomere.^8^ Despite the close position with other members of the albumin family,^9^ the multifunctional nature of VDBP is completely different from that of *ALB*, *AFP*, and *AFM*. Additionally, the *GC* gene is situated on chromosome 5 in mice and chromosome 14 in rats, both of which consist of 13 exons. Enhancing our understanding of the genomic structure and evolutionary background of the VDBP gene will expand our knowledge regarding its role in diverse physiological and pathological processes, including potential connections to psychiatric and neurological disorders.

### VDBP polymorphism

VDBP is known for its extraordinary genetic diversity, with more than 124 variant alleles documented, which positions it as one of the most highly polymorphic genes.^10^ The population has three frequently occurring VDBP structural polymorphisms that are linked to the polymorphisms rs7041 (T > G) and rs4588 (C > A). The two single nucleotide polymorphisms found in exon 11, which are situated in domain Ⅲ, are associated with the three primary VDBP variants: GC1F, GC1S, and GC2.^11^ GC1F and GC1S exhibit a disparity due to a solitary alteration in the amino acid composition at position 416, wherein aspartic acid is substituted with glutamine. Four amino acids (152 Gly → Glu, 311 Glu → Arg, 416 Asp → Glu, and 420 Arg → Thr) and the attached carbohydrates differentiate GC1 from GC2.^12^ GC1 and GC2 exhibit O-glycosylation, while GC1F and GC1S possess N-acetyl-d-glucosamine at their core.^13^^,^^14^ The distribution of these three isoforms varies significantly among various racial groups. GC1F is most common in populations of African descent, whereas GC1S is most common in Europeans, and Asians have intermediate frequencies of both GC1 forms. GC2 is rare in Black people and has a similar frequency in people of Asian or European descent.^15^ The differences in frequencies of VDBP isoforms may have important consequences for the metabolism of vitamin D and its related health consequences, as well as for the vulnerability to diseases in various populations.

### Protein structure of VDBP

VDBP is a type of α2-globulin with a weight of 52–59 kDa, and its precise weight is determined by its glycosylation state. VDBP, along with albumin, α-albumin, and α-fetoprotein, belongs to the albumin superfamily. The cysteines and disulphide bridges in this family are highly conserved. They also exhibit the characteristic of a modular structure with a triple-domain. The primary structure contains a notable amount of cysteine residues, which are evenly distributed with neighboring cysteine residues. The disulfide bonds were formed between cysteine residues located at the distal sites; thus, different functional domains were shaped. The 458 amino acids that make up the mature human VDBP are categorized into three structural domains, each with a specific function. The first domain includes amino acids 1–191 and has the characteristic 7 α-helical arrangement. The second domain is similar to the first one, with only one coil fold replacing the 7-helix structure from amino acids 192 to 378. In the third domain, only helices 1–4 are observed, comprising amino acids ranging from 379 to 458.^16^^,^^17^

The binding site for vitamin D consists of hydrophobic amino acids located in helices 1–6 (specifically amino acids 35–49) within the initial domain. In fact, only one binding site exists for all D metabolites. VDBP exhibits the greatest attraction towards 25OHD lactones, with 25OHD, 24,25(OH)2D, and 1,25(OH)2D following closely.^18^, ^19^, ^20^ The binding site of the vitamin D receptor is entirely distinct from that of VDBP to vitamin D. The N-terminal binding site of VDBP is a cleft situated on the vitamin D surface, while the vitamin D receptor-vitamin D binding site is a closed pocket within the receptor's internal structure.^16^ Only 1%–2% of VDBP binds to vitamin D,^21^ indicating that VDBP functions beyond its vitamin D transport properties. The actin-binding region was discovered within residues 373 and 403, present in both sections of domains Ⅱ and Ⅲ,^22^ and also in conjunction with a portion of domain Ⅰ.^23^^,^^24^ Additionally, the second and third domains of VDBP bind to fatty acids and extracellular structural proteins after cell necrosis or tissue damage.^25^^,^^26^ The C5a chemotactic cofactor activity was observed in a 20-amino-acid sequence located in the N-terminal domain I of VDBP, specifically residues 130–149.^27^ The binding site for C5a/C5a des Arg is located between amino acids 126 and 175, while the plasma membrane binding site was found between amino acids 150 and 172, as well as amino acids 379 and 402. Zhang et al identified two separate sequences (amino acids 150–172 in domain Ⅰ and amino acids 379–402 in domain Ⅲ) responsible for the binding of VDBP to cells, allowing VDBP to act as a connector protein, linking two separate molecules on the cell surface, which is a requirement for VDBP to carry out its cellular activities.^28^ The enzyme at the carboxyl terminus of VDBP can cut VDBP into the lower molecular weight VDBP-L (53.4 kD). Vitamin D binding protein-macrophage activating factor also known as DBP-L was observed to be a powerful activator of macrophages.^13^ The third domain contained the polymorphic changes of the three most prevalent genetic variants (GC1S/1F/2), whereas the second domain exhibited polymorphic differences caused by a few other variants.

### Synthesis, distribution, and measurement of VDBP

VDBP is synthesized and secreted predominantly by hepatic parenchymal cells in the liver. Nonetheless, the GC gene is also present in numerous tissues at minimal levels, including the kidney, testis, spleen, yolk sac, endocrine pancreatic cells, and adipocytes.^29^^,^^30^ The presence of VDBP *in situ* synthesis was reported in some brain regions, as well as in the spinal neurons and pia mater tissues of rats.^31^ The average daily production of VDBP for an adult individual is approximately 0.69–0.93 g/day, with a mean of 10.1 mg/kg/day.^32^^,^^33^ VDBP has a half-life of approximately 1.7 days in the plasma of humans. It is found in body fluids such as serum, urine, breast milk, ascites, cerebrospinal fluid (CSF), saliva, and semen. Additionally, VDBP has demonstrated an ability to engage with the surface of numerous cells through chondroitin sulfate proteoglycans. These cells include neutrophils, fibroblasts, monocytes, B and T cells, B lymphoblastoids, placental cytotrophoblasts, human sperms, smooth muscle cells, porcine kidney tubule cells, and rat pancreatic acinar cells.^34^ Serum concentration of VDBP ranges from 350 to 500 μg/L, with reduced levels found in other body fluids.^35^

The levels of VDBP in the serum fluctuate throughout the day, decreasing in the morning and then rapidly rising as the day progresses.^36^ Furthermore, a 5-fold disparity is observed in the average serum VDBP level among the three prevalent VDBP types (GC1F > GC1S > GC2), which could be attributed to variances in the synthesis or metabolic rate of VDBP in the different isoforms.^37^ VDBP production is not regulated by vitamin D itself or any of its metabolites.^38^ The production of VDBP is increased by dexamethasone and specific cytokines such as IL-6, while TGF-β reduces GC mRNA in a dosage-dependent manner.^39^ The use of birth control pills, body mass index, lipid parameters, and smoking were observed to be linked to both plasma and serum VDBP concentrations.^40^, ^41^, ^42^, ^43^

The VDBP concentration of body fluids was initially measured using radioimmunoassay, crossed immune-electrophoresis, rocket immunoelectrophoresis, and single radial immunodiffusion.^44^ Subsequently, nephelometry, turbidimetry, and enzyme-linked immunosorbent assay have been employed.^26^^,^^45^^,^^46^ Recently, liquid chromatography-tandem mass spectrometry was utilized to identify distinct VDBP proteoforms.^47^ Furthermore, innovative approaches in genomics, proteomics, and glycoproteomics have demonstrated the potential value of VDBP and vitamin D binding protein-macrophage activating factor across a broad range of applications.^48^, ^49^, ^50^ The VDBP concentration on the surface of cells can be measured by semi-quantitative methods such as immunoblotting and immunohistochemistry. In conclusion, measuring VDBP concentrations has evolved from traditional methods to more advanced techniques, allowing for a deeper understanding of its structural variants, proteoforms, and functions in health and disease. The diverse array of techniques available has broadened our knowledge of VDBP's involvement in a wide spectrum of biological processes.

### Biological function of VDBP

VDBP has many biological functions. The primary function of VDBP is to transport vitamin D, which binds to 85%–90% of the overall circulating calcidiol and has a notable role in preserving this homeostasis.^51^ Additionally, it attaches to actin subunits and has a vital function in depolymerizing extracellular actin filaments.^52^ It also contributes to the process of leukocyte C5a-mediated chemotaxis,^53^ the activation of macrophages,^3^ and the binding of the megalin–cubilin receptor.^54^^,^^55^ The involvement of VDBP in psychiatric and neurological disorders could potentially be associated with the aforementioned biological mechanisms.

### VDBP in psychiatric and neurological disorders

Numerous researchers have attempted to link VDBP expression with susceptibility or resistance to psychiatric and neurological disorders. Clinical studies and animal experiments have been conducted to explore the impact of VDBP on specific psychiatric and neurological conditions. Table 1, Table 2 offer a comprehensive overview of the changes in VDBP levels observed in the blood and CSF in psychiatric and neurological disorders, respectively. Additionally, Table 3 provides a summary of the association between VDBP polymorphisms and psychiatric and neurological disorders. These details have been elaborated in the subsequent sections.

**Table 1:** Overview of the expression of blood VDBP in psychiatric and neurological disorders.

Disease	Effect	Group characteristics	Reference
Depressive disorder	↑	74 patients with depressive disorder and 60 controls	Shi, 2020^49^
	↑	265 overweight/obese women	Maddahi, 2022^56^
Schizophrenia	–	42 patients with schizophrenia and 60 controls	Shi, 2020^49^
Bipolar depression	↑	685 youths	Petrov, 2018^66^
Alzheimer's disease (AD)	↑	20 patients with AD and 20 controls	Kułakowska, 2018^70^
	↑	10 patients with AD and 10 controls	Liao, 2007^71^
	↑	920 patients with AD, 277 patients with mild cognitive impairment, and 819 controls	Bishnoi, 2015^72^
	↓	261 patients with mild cognitive impairment, 24 patients with AD, and 411 controls from the Sydney Memory and Ageing Study; 180 patients with mild cognitive impairment and 153 controls from the Hunter Community Study	Muenchhoff, 2015^73^
Multiple sclerosis	–	42 patients with multiple sclerosis and 20 controls	Kułakowska, 2018^70^
Amyotrophic lateral sclerosis	↑	12 patients with amyotrophic lateral sclerosis and 20 controls	Kułakowska, 2018^70^
Meningitis	↑	16 patients with meningitis and 30 controls	Bahr, 2018^103^
Restless legs syndrome (RLS)	↑	7 patients with RLS and 6 controls	Shin, 2020^97^
	↓	12 patients with RLS and 10 controls	Mondello, 2021^98^
Migraine	–	52 patients with migraine and 49 controls	Celikbilek, 2014^102^

**Table 2:** Overview of the expression of VDBP in the cerebrospinal fluid in psychiatric and neurological disorders.

Disease	Effect	Group characteristics	Reference
Alzheimer's disease	↑	48 patients with Alzheimer's disease and 95 controls	Zhang, 2008^75^
	↓	10 patients with Alzheimer's disease and 10 controls	Abdi, 2006^76^
Parkinson's disease	↑	40 patients with Parkinson's disease and 95 controls	Zhang, 2008^75^
	↓	10 patients with Parkinson's disease and 10 controls	Abdi, 2006^76^
Multiple sclerosis (MS)	↓	12 patients with relapsing-remitting MS and 12 controls	Lehmensiek, 2007^81^
	↓	30 patients with relapsing-remitting MS and 36 controls	Liu, 2009^82^
	↓	10 patients with relapsing-remitting MS and 10 controls	Qin, 2009^83^
	↓	17 patients with relapsing-remitting MS and 17 controls	Kroksveen, 2012^84^
	↑	14 patients with clinically isolated syndrome, 45 with relapsing-remitting MS, 17 with secondary progressive MS, and 36 controls	Ottervald, 2010^85^
Amyotrophic lateral sclerosis	↑	12 patients with amyotrophic lateral sclerosis and 20 controls	Kułakowska, 2018^70^
Epilepsy	↑	15 patients with epilepsy and 15 controls	Xiao, 2009^50^
Restless legs syndrome	↑	5 patients with early-onset restless legs syndrome and 5 controls	Patton, 2013^96^

**Table 3:** Overview of the clinical implications of VDBP polymorphisms in psychiatric and neurological disorders.

Disease	Effect	Group characteristics	Reference
Depressive disorder	rs4588 rs7041-	330 patients with depressive disorder and 330 controls	Pillai, 2021^58^, 2022^59^
	rs4588 rs7041-	63 patients with depressive disorder and 1590 controls	Terock, 2020^60^
	GC2↑	121 patients with depressive disorder and 128 control	Pooyan, 2018^62^
	rs4588 rs7041-	5783 study participants	Terock, 2021^61^
Schizophrenia	GC1S↓	215 patients with schizophrenia and 199 controls	Papiha, 1982^64^
	GC2↑	423 patients with schizophrenia and 595 controls	Tsoi, 1990^65^
Autism spectrum disorder	GC1F↑	309 patients with autism spectrum disorder and 831 controls	Bolognesi, 2022^67^
Alzheimer's disease	–		Handy, 2021^69^ Zhang, 2020^74^
Parkinson's disease	rs7041(TT)↑	382 patients with Parkinson's disease and 242 controls	Gezen-Ak, 2017^79^
Restless legs syndrome	rs4588 rs7041-	285 patients with restless legs syndrome and 325 controls	Félix Javier, 2021^99^
Migraine	Variant (R21L)↑	4 patients with migraine and 10 controls	Nagata, 2014^100^
	rs4588 rs7041-	290 patients with migraine and 300 controls	García-Martín, 2023^101^

## Clinical implications of VDBP in psychiatric disorder

### Depressive disorder and VDBP

Depressive disorder, the most prevailing psychiatric condition, is characterized by enduring feelings of sadness, diminished interest, and a reduced sense of personal value. The association between serum VDBP levels and depressive disorder has been a subject of growing interest in recent years. Multiple epidemiological and clinical investigations have indicated that increased levels of serum VDBP are linked to a higher likelihood and greater intensity of depressive symptoms. Both plasma and postmortem dorsolateral prefrontal cortex tissues of patients with depressive disorder showed increased VDBP, as evidenced by a comprehensive analysis of plasma proteomics and verification in independent cohorts, along with a proteomic analysis of postmortem brain tissue.^49^ Another cross-sectional investigation revealed that women who had elevated levels of serum VDBP were more likely to experience a depressive disorder.^56^ A recent study by Zhang et al revealed that VDBP in plasma microglia-derived extracellular vesicles derived from plasma microglia could potentially be used as a biomarker for diagnosing depressive disorder.^57^ This finding offers valuable understanding regarding the mechanism of VDBP from the central brain to the periphery. Despite the strong correlation between VDBP and depressive disorder, the precise mechanisms linking the two remain uncertain and require further validation through additional experimental and clinical evidence.

Several research studies have indicated a favorable association between VDBP gene polymorphisms and depression risk and severity. Individuals with the mutation have a higher probability of experiencing and worsening depressive disorder. In a study involving 660 women, the VDBP variants rs7041 and rs4588, along with their haplotypes, did not have a significant association with postpartum depressive disorder susceptibility in the South Indian population. However, the presence of VDBP variants rs4588 and rs7041 was linked to lower levels of circulating serum vitamin D, which in turn was associated with an increased risk of postpartum depressive disorder.^58^^,^^59^ Another cross-sectional study involving more than 1000 individuals from Pomerania also revealed a positive correlation between the severity of post-traumatic stress disorder and VDBP polymorphisms (rs4588 and rs7041).^60^ However, a study on alexithymia, a characteristic of personality linked to different mental health conditions such as depressive disorder and post-traumatic stress disorder, indicates that no notable connection exists between the severity of the diseases and VDBP polymorphisms (rs4588 and rs7041).^61^

Moreover, the investigation of how dietary patterns and VDBP gene polymorphisms interact in terms of their impact on the risk of depressive disorder is a fascinating field of study. The role of diet is essential in regulating the availability and metabolism of VDBP. Since VDBP is responsible for transporting vitamin D, dietary factors have the potential to affect VDBP function and its connection to depressive disorder. A study conducted on 265 European adults discovered a connection between depression scores and the rs7041 and rs4588 polymorphisms. Additionally, a high-protein/low-fat diet and the rs7041 polymorphism were observed to have a remarkable impact on the moderate and severe depression groups. The study also suggested that a high-protein/low-fat diet could potentially mitigate the risk of depressive disorder in individuals with the VDBP genotype.^62^ The reason for this could be linked to the fact that high protein/low-fat diets elevate the available vitamin D levels in the serum, while high carbohydrate/low protein diets decrease the serum vitamin D levels.

Overall, the relationship between VDBP levels, gene polymorphisms, dietary patterns, and depressive disorder is complex and requires further investigation. Understanding these interactions may have implications for personalized approaches to depression management and treatment based on an individual's genetic makeup and dietary habits. Further investigation is necessary to understand the complex relationship between VDBP levels, gene polymorphisms, dietary patterns, and depressive disorder. Comprehending these interactions could potentially impact personalized strategies for managing and treating depressive disorder, considering an individual's genetic composition and dietary preferences.

### Schizophrenia and VDBP

Schizophrenia is another prevalent and complex psychiatric disorder with an uncertain etiology. Research has focused on the possible connection between schizophrenia and VDBP deficiency or abnormality, although the findings on the correlation between VDBP levels and schizophrenia are not completely uniform. Although some proteomic studies have shown no significant difference in VDBP plasma levels in patients with schizophrenia compared with normal controls,^49^ additional research suggests that the phosphorylation of the VDBP protein in the serum is reduced in individuals with schizophrenia following olanzapine treatment,^63^ suggesting a possible association between schizophrenia and VDBP levels. VDBP polymorphisms have been found to be associated with the onset of schizophrenia in certain studies. Female patients were observed to exhibit notably reduced frequencies of the GC1S allele among the subtype allele frequencies, indicating a variation in vulnerability to the GC motif based on sex.^64^ According to research on Chinese individuals with schizophrenia, the susceptibility to this condition is associated with the presence of GC2, whereas GC1S provides a protective effect against schizophrenia.^65^ Nevertheless, schizophrenia must be considered a highly complex and multifactorial disorder with a combination of genetic, environmental, and neurobiological factors contributing to its development. The role of VDBP in schizophrenia may be just a single piece of an intricate puzzle.

### Other psychiatric disorders and VDBP

Few other psychiatric disorders have been reported to correlate with VDBP. A study discovered that the levels of VDBP in the serum of individuals with bipolar depression were considerably elevated compared with that of those in the non-mood control group.^66^ VDBP plays a role in the pathogenesis of bipolar depression in adolescents, or VDBP is a factor associated with this disorder. A different study uncovered the correlation between autism spectrum disorder and genetic variations in the VDBP gene. The GC1F genotype is significantly more likely to be observed in children with autism spectrum disorder. The occurrence of the GC1F variant, whether in the homozygous or heterozygous state, was discovered to be linked to heightened clinical symptoms of autism spectrum disorder and decreased overall functioning.^67^ Yee et al observed that a comparative analysis of 31 individuals experiencing their initial episode of psychosis and exhibiting symptoms such as positive syndrome, negative syndrome, excitement, depression, or cognitive impairment, showed elevated levels of VDBP in contrast to controls.^68^

Overall, these studies suggest that VDBP may be involved in the pathogenesis of various psychiatric disorders and its role deserves further in-depth investigation. However, psychiatric disorders are highly complex multifaceted conditions with a multitude of factors contributing to their development. VDBP is merely one of the numerous possible factors that might contribute to these disorders, and a thorough investigation is necessary to understand its specific mechanisms in each case. Further research in this area may offer valuable insights into the pathophysiology of psychiatric disorders and potentially lead to the development of new therapeutic strategies.

## Clinical implications of VDBP in neurological disorder

### AD and VDBP

AD is characterized by progressive memory and cognitive decline, along with neuronal loss in the brain's cortical and hippocampal areas and the formation of amyloid beta protein plaques and neuroprogenitor fiber tangles. The correlation between VDBP polymorphisms and AD is a subject of continuous investigation and the results have shown discrepancies across different research studies.^69^ The existence of alterations in the VDBP levels in the blood or the CSF of individuals with AD is unclear. Several research studies indicated that the concentration of VDBP in their blood plasma was notably elevated in comparison to that of controls,^70^^,^^71^ and serum VDBP levels have a strong positive correlation with a latent dementia phenotype.^72^ While some other studies have found that VDBP was significantly decreased in participants with mild cognitive impairment,^73^ VDBP levels may contribute to slowing down the cognitive decline and forestalling AD.^74^ The controversial nature of the changes to VDBP in the CSF of patients with AD is also a subject of debate. Several studies showed that the levels of VDBP collected from patients with AD were notably elevated compared with that of the controls,^70^^,^^75^ whereas other studies did not find any notable differences.^76^ These inconsistent results may be attributed to various factors, including the sample size, the baseline characteristics, the methods used in the experiment, and the genetic background. Some studies have demonstrated direct interactions between purified VDBP and amyloid beta protein in animal tests. VDBP was discovered to attenuate amyloid beta protein aggregation and accumulation, decrease the loss of synapses caused by amyloid beta protein in the hippocampus, and restore memory impairments in mice.^77^^,^^78^ These findings suggest that VDBP may have a potential therapeutic role in AD treatment.

### PD and VDBP

The role of VDBP in PD has received less attention compared with other neurological disorders. Nevertheless, several research studies have explored the correlation between VDBP and PD, yielding interesting but occasionally contradictory findings. One study observed a higher occurrence of rs7041 polymorphisms among all patients diagnosed with PD.^79^ It is also linked to essential tremor, which is clinically identified by the existence of postural and kinetic tremor that impacts the upper limbs.^80^ Zhang et al found that the increase in VDBP in the CSF was more noticeable in PD compared with AD,^75^ while Abdi et al discovered that the expression of VDBP in the CSF of individuals with PD was lower when compared with that in the controls.^76^ The variations in the manifestation of VDBP in the CSF of individuals with PD might be associated with variances in ethnic background, disease stage, medication usage, and other factors. The pathogenesis of PD is complex and likely involves a combination of genetic, environmental, and neurobiological factors. VDBP, being a multifunctional protein with diverse roles, may have varying impacts depending on disease context and individual characteristics. The changes in VDBP in PD, its association with the clinical features, and its molecular mechanisms warrant further investigation and validation through additional research. Large-scale studies conducted on diverse populations and longitudinal assessments could provide further insights into the importance of VDBP in PD and its potential implications for the development and management of the disease. As with other neurological disorders, understanding the role of VDBP in PD is assumed to be an intricate and continuous procedure.

### MS and VDBP

MS is a predominant demyelinating disorder affecting the central nervous system. An increasing amount of supporting research backs the importance of VDBP in MS. Irregular VDBP levels in the blood or spinal fluid of individuals with MS might be linked to the likelihood of developing MS, the display of clinical symptoms, and the progression of the disease. The status of VDBP levels in the CSF of patients with MS is controversial. Notable findings have been produced through research aimed at identifying biomarker proteins that are specifically up-regulated or down-regulated in the CSF of patients with various stages of clinically isolated syndrome and MS. Lehmensiek et al showed a reduction in VDBP levels in the CSF collected during an acute relapse in patients with clinically isolated syndrome, though not in patients with relapse-remitting MS.^81^ Subsequent studies have demonstrated reduced VDBP levels in the CSF of individuals with relapsing-remitting multiple sclerosis during a relapse, in contrast to that of individuals with other neurological disorders.^81^, ^82^, ^83^, ^84^ Nevertheless, a proteomics-based biomarker discovery study of 209 individuals revealed that the concentrations of VDBP were elevated in the CSF of patients with relapsing-remitting multiple sclerosis and those with secondary progressive MS.^85^ The lack of consistency may be attributed to potential confounding factors such as variations in sample size, ascertainment criteria, timing of CSF sampling, and analysis methods. During the acute relapse of MS, the CSF exhibited lower VDBP levels, whereas during the progressive phase, higher levels were observed.

Multiple studies have demonstrated that VDBP polymorphisms (rs7041 and rs4588) are not associated with the susceptibility to MS. Several studies, including a Sicilian case–control study, a Japanese case–control study, a family-based and a candidate gene study of Canadian patients with MS, and an American nested case–control study, did not discover any connection between VDBP phenotypes and the occurrence of MS.^86^, ^87^, ^88^, ^89^, ^90^ Hence, the presence of disease, disease natural history, or the age of onset is not significantly affected by VDBP single nucleotide polymorphisms. The involvement of VDBP in MS is intricate and the variations in the results can be ascribed to various factors, such as the diversity of the illness itself, distinct study cohorts, and constraints of the research techniques employed. Additional investigation is required to clarify the precise function of VDBP in the development of MS and its possible application as a biomarker or target for the treatment of the condition.

### ALS and VDBP

An association between ALS and VDBP has been observed. The healthy controls did not show the presence of GC2, according to a plasma proteomic analysis conducted on a group of Portuguese patients with familial ALS. Simultaneously, a reduction in the presence of more acidic variants of VDBP was observed among the individuals with familial ALS. The findings indicated that the GC2 variation of VDBP might pose a hazard for ALS, potentially resulting in heightened vulnerability to the progression of the disease in patients with ALS.^91^ Furthermore, higher levels of VDBP were observed in the blood plasma and CSF of patients with ALS by enzyme-linked immunosorbent assay.^70^ The results offer valuable perspectives on the possible involvement of VDBP in ALS and emphasize the necessity for additional studies to gain a better comprehension of its distinct impact on the mechanisms of the disease.

### Epilepsy and VDBP

VDBP is also associated with seizures and is elevated in the CSF of patients with temporal lobe epilepsy according to proteomic analysis.^50^ The presence of increased VDBP in the CSF might indicate brain injury caused by epileptic seizures. Pathological alterations in the structure and functioning of neurons caused by seizures, such as neuronal death, programmed cell death, and reduction in dendritic spines, cause the release of G-actin into the surrounding space. Consequently, an escalation occurs in VDBP binding to G-actin.^92^^,^^93^ Additionally, VDBP polymorphisms have demonstrated a correlation with vulnerability to epilepsy. A study involving 430 participants showed that the polymorphisms of VDBP rs4588 may play a potentially important role in the vulnerability to epilepsy in the Chinese Han community.^94^ The over-expression of VDBP in the hippocampus was also demonstrated to increase vulnerability to kainate-induced seizures in rats. VDBP could potentially serve as a notable transcriptional connection between hippocampal activation and neuroplasticity.^95^ The combined findings of these studies indicate that VDBP and its polymorphisms might play a role in the development of seizures and epilepsy.

### RLS and VDBP

RLS, a complex sensorimotor disorder characterized by an uncontrollable urge to move the legs and abnormal sensations, has been the subject of several studies examining its relationship with VDBP. A proteomic analysis of CSF in a limited sample size (five early-onset patients with RLS *vs*. five controls) revealed elevated levels of CSF VDBP in individuals with RLS.^96^ Two studies investigating changes in serum VDBP levels in RLS yielded conflicting results.^97^^,^^98^ One study comparing seven patients with RLS to six matched controls demonstrated a significant up-regulation of VDBP in individuals with RLS, while another study involving 12 patients with RLS and 10 matched controls reached a different conclusion. The inconsistency in the direction of association can be attributed to crucial methodological variations, timing of sample collection, and limited sample size between the studies. Additionally, a study conducted genotyping on 285 idiopathic patients with RLS and 325 controls in the Spanish Caucasian population for the VDBP variants rs7041 and rs4588, and it discovered no association between VDBP polymorphisms and RLS.^99^ The preliminary research on the correlation between VDBP and RLS provides a foundation for future exploration, paving the way for a thorough investigation into the mechanistic role of VDBP in the progression of RLS.

### Migraine and VDBP

Migraine is a prevalent and chronic neurological disorder characterized by severe headaches and dysfunction of the autonomic nervous system. Nagata et al conducted linkage analysis and exome sequencing in a family with four affected individuals and discovered a variant (R21L) in exon 2 of VDBP, which affected cytokine release, thereby affecting migraine.^100^ However, a study that genotyped 290 patients diagnosed with migraine and 300 matched controls suggested no association between the polymorphisms of VDBP single nucleotide polymorphisms and the risk of developing migraine.^101^ Similarly, another cross-sectional prospective study with 52 patients with migraine and 49 controls found that serum VDBP levels did not correlate with migraine or headache characteristics, including aura, attack severity/frequency/duration, and disease duration.^102^ Given the current state of research, evidence is scarce to establish a direct relationship between VDBP and migraine. The present study suggests that more in-depth research is necessary to conclusively determine whether such a direct relationship exists.

### Other neurological disorders and VDBP

Despite the limited research on VDBP and meningitis, studies have revealed that patients with tuberculosis meningitis have a considerably higher average VDBP concentration compared with individuals without. Additionally, VDBP has demonstrated satisfactory diagnostic capabilities for identifying tuberculosis meningitis.^103^ A large follow-up study found that individuals with deficient levels of 25(OH)D, genetically predisposed to high VDBP polymorphisms (rs4588 and rs7041), might face an increased susceptibility to stroke.^104^ Additionally, VDBP has been detected in the rat brain and has been shown to stimulate neuronal transcription of the tissue-type plasminogen activator, leading to an increase in N-methyl-d-aspartate-mediated neurotoxicity.^105^ This implies that VDBP might influence neurotoxic mechanisms and potentially contribute to the pathogenesis of specific neurological disorders.

Although extensive research is lacking in these fields, the results suggest that VDBP could potentially impact the development and identification of specific neurological conditions such as meningitis, stroke, and neurotoxicity. Nevertheless, further investigation is needed to comprehensively understand the fundamental mechanisms and clinical implications of these associations.

## Conclusions and future perspectives

The current research indicates that the levels of VDBP and genetic polymorphisms are linked to a range of psychiatric and neurological conditions. Nevertheless, disparities remain in the findings, and further investigations are required to clarify the mechanisms that underlie these connections. Investigating VDBP in the CSF and serum can offer valuable insights into the pathogenesis of these disorders. VDBP has the potential to serve as a biomarker, offering hope for enhancing diagnosis, prognosis, and treatment approaches. Additionally, understanding the precise mechanisms by which VDBP acts in the nervous system could provide important clues for developing targeted therapies. In the future, research should focus on clarifying the functional significance of VDBP in different neurological and psychiatric conditions. Large-scale studies with diverse populations and well-defined cohorts are needed to validate the findings and establish robust associations. Moreover, exploring the interactions between VDBP and other biological factors, such as vitamin D, cytokines, and genetics, can deepen our understanding of its complex role in these disorders. Overall, the study of VDBP in psychiatric and neurological disorders represents an exciting and promising area of research. Persisting endeavors in this domain could potentially result in innovative treatment approaches and enhanced results for individuals impacted by these intricate and demanding ailments and their complex and challenging conditions.

## Conflict of interests

The authors declared no conflicting concerns regarding the described work.

## Funding

This project was supported by grants from the National Natural Science Key Foundation of China (No. 82130042 to ZJ Zhang) and the 10.13039/100014717National Natural Science Foundation of China (No. 82371532 to Y Kong).
